# Integrative analysis of genome and transcriptome reveals a novel regulator for pork intramuscular fat content

**DOI:** 10.1186/s12711-025-01014-9

**Published:** 2025-11-06

**Authors:** Xueyan Zhao, Jingxuan Li, Wanli Jia, Yifan Ren, Yanping Wang, Tizhong Shan, Jiying Wang

**Affiliations:** 1 Institute of Animal Science and Veterinary Medicine, Shandong Academy of Agricultural Sciences, Jinan, 250100 People’s Republic of China; 2 Key Laboratory of Livestock and Poultry Multi-omics of MARA, Jinan, 250100 People’s Republic of China; 3 Shandong Provincial Key Laboratory of Livestock and Poultry Breeding, Jinan, 250100 People’s Republic of China; 4 Zhejiang University, Hangzhou, 310058 People’s Republic of China

## Abstract

**Background:**

Pork is a primary source of animal protein worldwide, and intramuscular fat (IMF) content is a key determinant of meat quality and consumer preference. To identify genetic regulators of IMF content, we leveraged RNA sequencing and whole-genome resequencing data from 79 Laiwu pigs renowned for high IMF content to conduct expression quantitative trait locus (eQTL) mapping. We integrated eQTL results with genome-wide association study (GWAS) data from 453 Chinese Lulai Black pigs (a crossbreed of Laiwu and Yorkshire pigs), and systematically identified candidate regulatory genes for IMF content by incorporating weighted gene co-expression network analysis (WGCNA) and correlation analysis in this population.

**Results:**

We identified 9,763 cis-eQTLs at the genome-wide level (*p* < 5E−08) and 1,337 cis-eQTLs at the suggestive level (*p* < 5E−06). A 2.02 Mb cis-QTL region on Sus scrofa chromosome 9, containing 587 cis-eQTLs regulating *MED17* expression, overlapped with an IMF-associated QTL detected by GWAS in Lulai Black pigs, a Laiwu-Yorkshire crossbreed. WGCNA identified three critical co-expression modules related to IMF content, with *MED17* acting as a critical gene in a module linked to adipogenesis and lipid metabolism. Correlation analysis showed *MED17* expression was negatively correlated with IMF content (FDR = 1.58E−02). In 3T3-L1 preadipocytes, adenovirus-mediated *Med17* overexpression significantly reduced adipogenic differentiation and altered expression of adipogenesis-related genes (*Pparg*, *Adipoq*, *Srebf1*, *Cpt1a*, and *Atgl*), indicating that *Med17* modulates adipocyte differentiation and lipid metabolism.

**Conclusions:**

This study identifies *MED17* as a novel regulator of IMF content in pigs, bridging genomic variation, gene expression networks, and phenotypic traits. These findings provide mechanistic insights into IMF deposition and highlight the potential of integrative multi-omics approaches for genetic improvement of pork quality traits in breeding programs.

**Supplementary Information:**

The online version contains supplementary material available at 10.1186/s12711-025-01014-9.

## Background

With the global economy burgeoning and the population expanding, the worldwide consumption of meat is on an upward trajectory. Among various types of meat, pork stands out as a vital source of high-quality protein in the human diet, accounting for approximately one-third of total meat consumption annually [1]. The escalating demand for pork products has spurred the expansion of the pig industry. In response to this growth, for decades, breeders have focused on selectively breeding pigs for higher growth rates and increased lean meat content, all with the ultimate goal of boosting pork production. This has led to a decline in pork quality traits, thereby affecting consumer preferences and meat competitiveness.

Adipose tissue provides important sensory properties and nutritional value to meat products. With decrease of adipose, intramuscular fat (IMF) content, a crucial factor for meat quality, has significantly decreased due to the emphasis on enhancing growth and meatiness in pigs. Currently, commercially produced pigs have an IMF content of approximately 1% [2], which falls below the recommended range of 2.2–3.4% for good taste [3]. Therefore, identifying genes that regulate IMF content and elucidating their regulatory mechanisms are essential for increasing IMF content and improving pork quality.

Many studies have been carried out to identify the critical regulators that affect the IMF content. According to PigQTLdb (https://www.animalgenome.org/cgi-bin/QTLdb/SS/index), 791 quantitative trait loci (QTL) associated with IMF content have been identified through QTL linkage analysis and genome-wide association studies (GWAS) as of Aug 2024. While these QTL can explain phenotypic variation to some extent, they do not fully elucidate the underlying molecular mechanisms governing IMF deposition. However, expression QTL (eQTL) studies offer valuable insights into gene regulation and relationships between phenotypes and genotypes. Therefore, integrating eQTL analysis and QTL mapping through GWAS represents a powerful approach for revealing the genetic architecture of complex traits in pigs. Higgins et al. [4] demonstrated the effectiveness of this integrative approach and identified a single nucleotide polymorphism (SNP) in *GFRA2* that influences residual feed intake in beef cattle. Moreover, based on GWAS and eQTL mapping results, Liu et al. [5] further combined quantitative trait transcript (QTT) analysis and weighted gene co-expression network analysis (WGCNA) to identify candidate genes affecting meat quality traits in pigs. For the IMF content trait, Liu et al. [6] performed an integrated analysis combining GWAS, eQTL mapping, allele specific expression (ASE), and IMF-gene correlation analysis using RNA-sequencing (RNA-seq) and BeadChip data in Duroc × Luchuan crossed pigs.

Laiwu pigs, a fat-type Chinese indigenous breed, are renowned for their exceptionally high IMF content, with levels reaching 10% to 13%—surpassing most other local Chinese pig breeds [7]. This remarkable trait makes them an excellent genetic resource for improving meat quality in modern commercial pigs. Lulai Black pigs were developed through a systematic breeding program involving crossbreeding, horizontal fixation, successive generation selection, and directional cultivation to leverage the complementary genetic advantages of Laiwu and Yorkshire pigs, yielding a crossbreed with about 62.5% Laiwu genetic ancestry. Specifically designed to balance meat quality with growth efficiency, this breed serves as a high-quality alternative to the Duroc × (Landrace × Yorkshire) three-way commercial cross. Their genetic admixture creates a unique population for mapping IMF-associated loci, where segregating variants from both parental breeds enhance QTL detection power to facilitate identification of key meat quality regulatory genes. Hence, we previously identified several SNPs and candidate genes associated with IMF content through a GWAS using GeneSeek Genomic Profiler Porcine HD BeadChip SNP data from 453 Lulai Black pigs [8]. Based on these results, this study integrates data of eQTL mapping, WGCNA and correlation analysis, to detect critical genes underlying high IMF content in Laiwu pigs. While previous studies, including Liu et al. [6], have integrated transcriptomic and genomic data, the challenge of identifying genes with consistent cross-dataset support has persisted. In contrast, our multi-omics approach uniquely pinpointed *MED17* through rigorous cross-validation across integrated omics layers. This finding was further validated via functional assays in 3T3-L1 cells, demonstrating *MED17*’s role in adipocyte differentiation. These findings not only unravel the role of *MED17* as a novel regulator of IMF in adipocyte differentiation but also highlight how integrative multi-omics bridges genetic variation, transcriptomic regulation, and phenotypic outcomes, laying a methodological foundation for marker-assisted selection in pig breeding to enhance meat quality.

## Methods

### Animal sampling and phenotyping

A total of 79 Laiwu pigs (58 males and 21 females) were collected from Laiwu pig conservation farms or breeding farms in Laiwu District of Jinan City, Shandong Province, China. These pigs were fed formulated diets with similar nutrient content according to the regional standards for Laiwu pig nutrient recommendations in China (DB37/T 3672-2019) and had free access to water. At approximately 100 kg body weight, the pigs were slaughtered in 11 batches following stunning by electric shock in the same slaughterhouse. Approximately 0.2 g of *Longissimus thoracis* (LT) tissue were collected from the last fourth and fifth thoracic vertebrae for IMF content examination and genomic RNA extraction. IMF content was measured using the Soxhlet petroleum ether method with approximately 200 g of LT muscle, following previously described protocols [8]. All experimental procedures were approved by the Institutional Animal Care and Use Committee of the Institute of Animal Husbandry and Veterinary Medicine, Shandong Academy of Agricultural Sciences (approval code: IACC20060101; January 1, 2006).

### eQTL mapping

Genomic DNA was extracted from the ear tissues of the 79 Laiwu pigs using a traditional phenol–chloroform extraction method, and genotyping was performed on an Illumina HiSeq instrument (See Additional file 1, Text S1). Individuals with an individual missing rate > 0.1 were excluded, and SNPs were excluded if they had a minor allele frequency < 0.05, SNP missing rate > 0.1, or were located in mitochondria and scaffolds. The remaining SNPs were used for further eQTL analysis. Total RNA extracted from the LT muscles was sequenced on an Illumina NovaSeq platform (See Additional file 1, Text S2). Before conducting the eQTL analysis, expressed gene normalization was performed based on the rank of transcripts per kilobase per million mapped reads (TPM) values across all samples.

Associations between SNPs identified through whole-genome resequencing and gene expression levels were identified using the R package Matrix eQTL [9]. The following an additive linear model was employed:

1 $$g\, = \,\beta \, * \,S\, + \,{\text{bw}}\, + \,{\text{se}}\, + \,{\text{ba}}\, + \,\varepsilon$$

In Eq. (1), *g* represents the normalized expression level of the target gene, *β* is the effect of the SNP allele substitution; *S* denotes the SNP genotype covariate (coded as 0, 1, and 2 for homozygotes for the reference allele, heterozygotes, and homozygotes for the alternative allele, respectively); bw represents carcass weight; se denotes sex; ba stands for slaughter batch; and ε is random residual error.

Tests were conducted individually for each gene-SNP pair, with a false discovery rate (FDR) calculated to control for multiple comparisons. Similar to the thresholds used in the GWAS based on genome resequencing data, the significance thresholds were defined as *p* < 5E−08 (FDR < 0.0064) for genome-wide significant associations, and *p* < 5E−06 (FDR < 0.0879) for suggestive level associations. SNPs that were one megabase (Mb) up- or downstream of the transcription start site of the target gene were designated as local cis-eQTLs, whereas the others were classified as trans-eQTLs.

### Co-localization of phenotypic QTLs (pQTLs) associated with IMF content with eQTLs

Gene expression profiles and regulatory mechanisms vary considerably between pig breeds. To identify pQTLs associated with IMF content, we utilized the pigQTLdb, entering “Laiwu” as a keyword and selecting “intramuscular fat content” as the trait of interest. Additionally, our previous GWAS on IMF content was conducted in a cohort of 453 Chinese Lulai Black pigs, which are derived from crossbreeding Laiwu and Yorkshire pigs and selectively bred for over eight generations [8]. The pQTL detected by this GWAS is not included in the pigQTLdb and was incorporated here as an additional source of pQTLs. For consistency with the cis-eQTL analysis, the coordinates of GWAS SNPs genotyped using GeneSeek Genomic Proffler Porcine HD BeadChip (Neogen Corporation, Lansing, MI, USA) based on the Sscrofa10.2 reference genome were converted to the Sscrofa11.1 reference genome using the UCSC LiftOVER tool. pQTL intervals were determined using the 2–LOD drop method, where one unit of −log_10_(*p*-value) approximates one unit of the logarithm of the odds (LOD) value. All SNPs on each chromosome with a LOD score higher than the peak LOD score (−log_10_(*p*-value)) minus 2 were retained [10].

### Construction of co-expression gene network

The WGCNA was constructed using the WGCNA package in R [11], as detailed in Additional file 1, Text S3. Modules with module-trait relationships (MTR) *p* value < 0.01 were deemed biologically significant. The genes within these modules were subjected to functional enrichment analysis using the DAVID database. Gene ontology (GO) terms and Kyoto Encyclopedia of Genes and Genomes (KEGG) pathways with *q* < 0.05 (adjusted *p*-value by the Benjamini–Hochberg method) were considered significant. Modules significantly enriched in IMF-related GO terms and pathways were identified as potentially critical. In addition, within these critical modules, genes with *p* values of both gene significance (GS) and module membership (MM) below 0.01 were considered critical. A protein–protein interaction (PPI) network for these critical genes was generated using STRING 12.0 (See Additional file 1, Text 4).

### Correlation between gene expression and IMF content

The IMF content and log_2_-transformed gene expression data value were corrected using a linear model, where sex and slaughter batch were incorporated as fixed effects, and carcass weight was used as a covariate. Pearson correlation coefficients were then calculated between the residuals of the log_2_-transformed expression levels and the corrected IMF content values using the “cor.test” function in R. The significance of these correlations was determined using the “p.adjust” function via the FDR method, with *q* ≤ 0.05 set as the significance threshold.

### Adenovirus-mediated overexpression of *Med17* in 3T3-L1 cells

The role of *MED17* in fat deposition was investigated in the 3T3-L1 murine preadipocyte cell line. Given the negative correlation between *MED17* expression and IMF content, we overexpressed mouse *Med17* in 3T3-L1 cells using adenovirus. The adenovirus expressing the *Med17* gene (Med17-oe) was constructed by WZ Biosciences, Inc (Jinan, China) followed the procedure detailed in Additional file 1, Text S5.

For adenovirus transfection, 3T3-L1 cells were cultured to 50% confluence and then transfected with *Med17*-expressing adenovirus at a multiplicity of infection (MOI) of 250 (See Additional file 1, Text S6). Cells transfected with the control adenovirus were treated identically to serve as controls (See Additional file 1, Text S5). The relative expression levels of *Med17* in cells infected with adenovirus for 48 h were detected by quantitative real-time PCR (qRT-PCR) analysis and western blot assay (See Additional file 1, Text S7 and Text S8). After eight days of adipogenic differentiation (See Additional file 1, Text S6), lipid accumulation in 3T3-L1 cells was evaluated using the Oil Red O Staining and total triglyceride (TG) assay (See Additional file 1, Text S9). Meanwhile, the expression of some genes associated with adipogenesis were detected in 3T3-L1 cells which were infected with adenovirus for 48 h and were subsequently differentiated for eight days (See Additional file 2, Table S1 and Additional file 1, Text S7).

### Statistical analysis

Data were presented as means ± standard errors (SE), derived from at least three independent experiments. Statistical analyses were performed using GraphPad Prism 9.0 software. Comparisons between two groups were performed using an unpaired two-tailed Student’s *t*-test. Statistical significance was defined as a *p*-value < 0.05, with *, **, and *** denoting *p* < 0.05, *p* < 0.01, and *p* < 0.001, respectively.

## Results

### Summary statistics of phenotypes and sequencing datasets

**Fig. 1:**
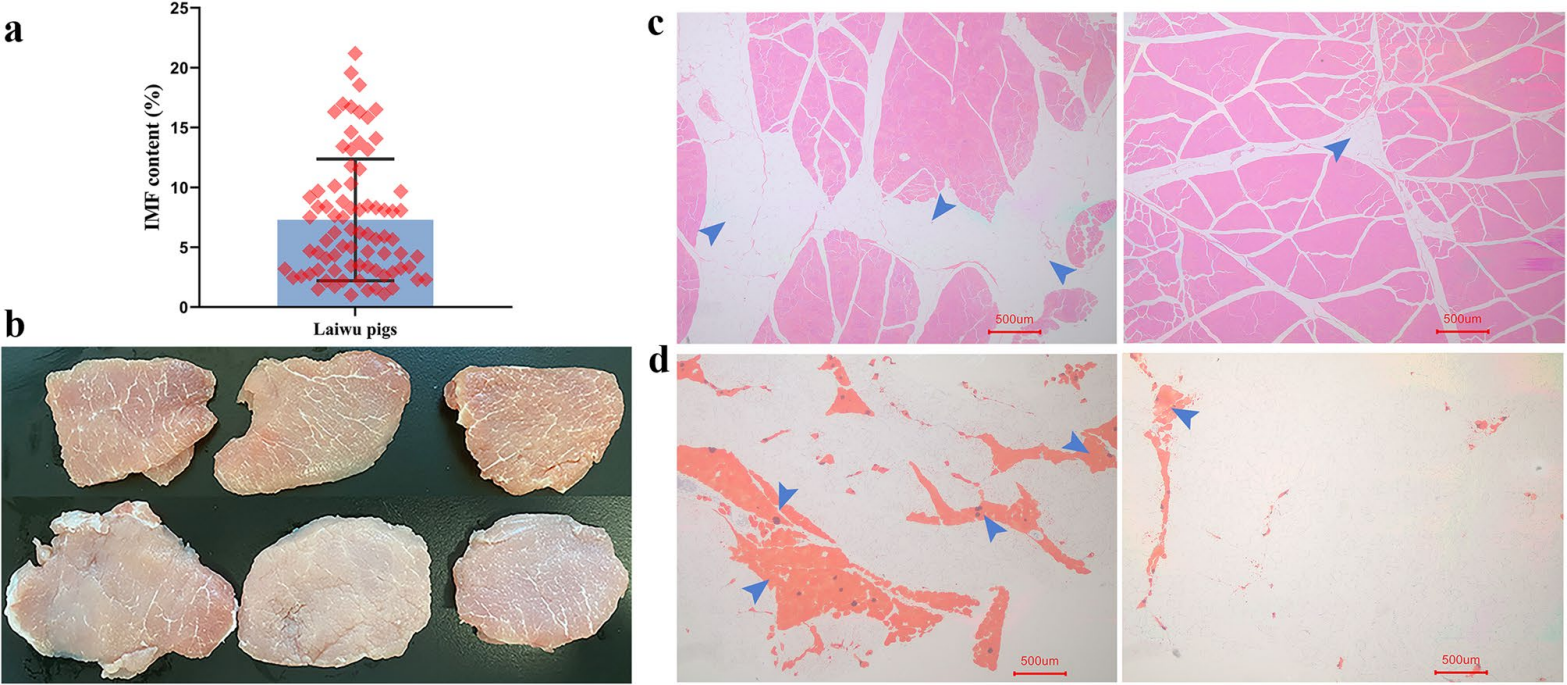
The intramuscular fat (IMF) content of Laiwu pigs. **a** The box-plot of IMF content in 79 Laiwu pigs. **b** The *Longissimus thoracis* (LT) tissues of Laiwu pigs. **c** Hematoxylin–Eosin (HE) staining of tissue LT sections from individuals with relatively high and low IMF content. **d** Oil Red O staining of tissue LT sections in individuals with relatively high and low IMF content. The blue arrows in figures (**c**) and (**d**) represent IMF

In this study, 79 Laiwu pigs were selected to determine IMF content. The average carcass weight of these pigs was 71.20 ± 6.53 kg. Notably, significant variability in IMF content was observed among individuals, spanning a range from 1.03% to 21.19%, with an overall average of 7.27% (as depicted in Fig. 1a and Additional file 2, Table S2). Differences in IMF content could also be found in the marbling of LT muscle (Fig. 1b). Subsequently, Hematoxylin–Eosin (HE) staining and Oil Red O staining were performed on LT muscle sections of a subset of individuals. After HE staining, fat manifested as small, pale red vacuoles dispersed throughout the muscle tissue (Fig. 1c). In contrast, when subjected to Oil Red O staining, the fat was distinctly stained red (Fig. 1d). The results of these stained sections effectively corroborated the IMF content values obtained through the Soxhlet petroleum ether extraction method.

Genomic DNA was isolated from Laiwu pig ear tissues for whole-genome resequencing. On average, 188.15 million sequence reads were generated, with an average read depth of 10.85-fold. After quality filtering, 186.61 million clean reads were obtained, with 98.66–99.64% of these reads successfully mapped to the Sus Scrofa11.1 reference genome (See Additional file 2, Table S3). Subsequently, variants calling and stringent quality filtering were carried out, resulting in the retention of 29,924,033 high-quality SNPs from 79 individuals for further eQTL detection.

RNA was extracted from 79 Laiwu pig LT muscle samples and sequenced using a paired-end RNA-seq approach on the Illumina NovaSeq platform. An average of 43,600,998 clean reads, each 150 bp in length, were produced, with 95.07% mapping to the reference genome and 91.82% mapping uniquely (See Additional file 2, Table S4). The mapped reads of each sample were assembled using StringTie with a reference-based approach, and the expression levels of 25,640 genes were quantified using FeatureCounts. Of these genes, 1,274 were novel. For further analysis, 14,588 genes were retained, each with Fragments Per Kilobase of exon model per Million mapped fragments (FPKM) value greater than 0.1 in more than half of the samples.

### Cis-eQTLs identified in Laiwu pigs

**Fig. 2:**
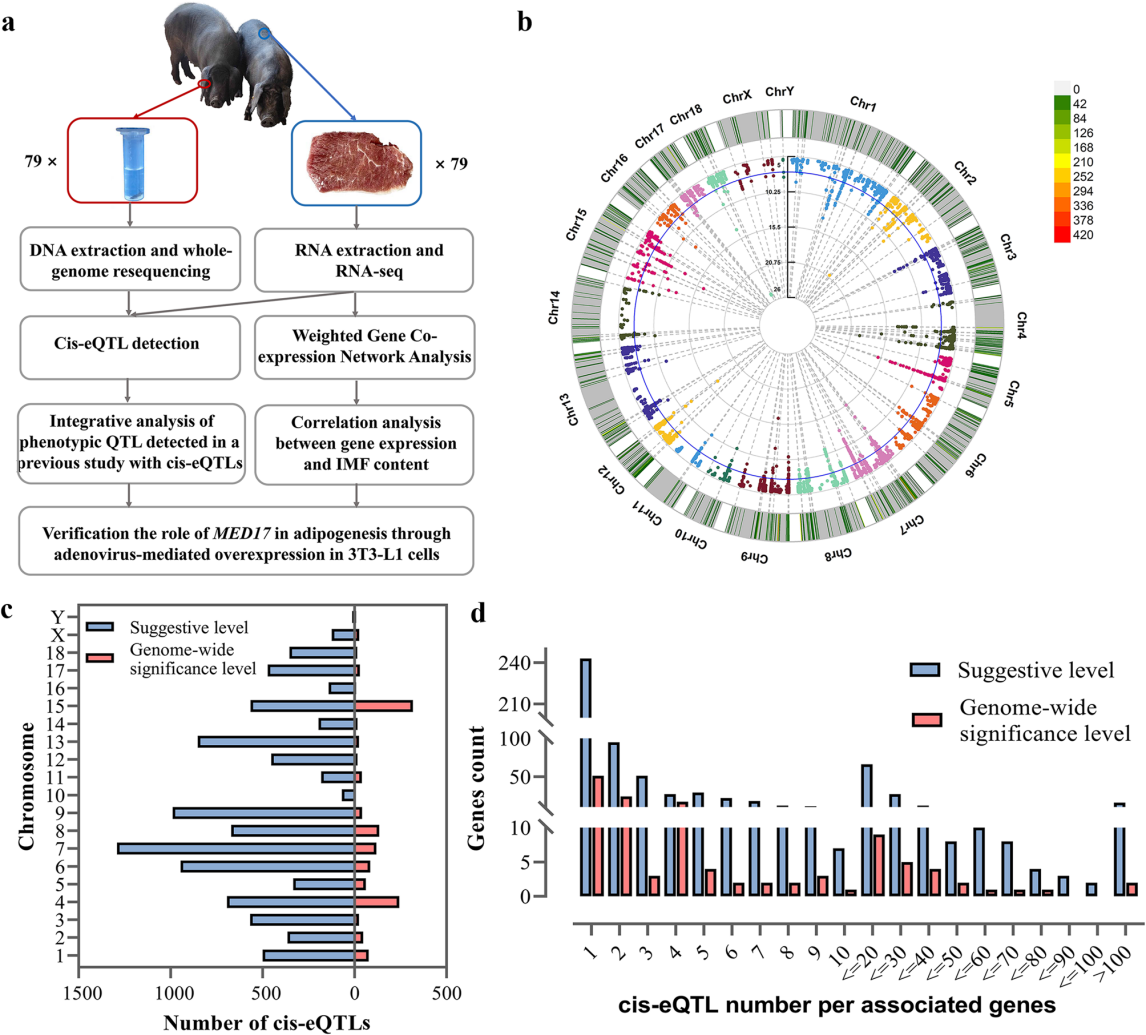
Cis-expression quantitative trait loci (cis-eQTL) identified in the *Longissimus thoracis* muscles of Laiwu pigs. **a** A schematic representation of the study design and analysis steps. **b** The CMplot of significant cis-eQTLs (*p* < 5E−06). The dots representing significant cis-eQTLs are color-coded based on their chromosome locations. Chr denotes chromosomes. The radius axis represents the negative base 10 logarithm of *p*-values, and blue circle crossing it represents the threshold of genome-wide level (*p* < 5E−08). The outer circle represents the distribution density of significant eQTLs on each chromosome. **c** The distribution of significant cis-eQTLs (*p* < 5E−06) on each chromosome. **d** The distribution of cis-eQTL-associated genes affected by varying numbers of cis-eQTLs

The study design and analytical framework are shown in Fig. 2a. To explore the relationship between SNPs and gene expression levels in Laiwu pigs, eQTL mapping was conducted. A total of 1,337 cis-eQTLs were identified at the genome-wide level (*p* < 5E−08) and 9,763 cis-eQTLs were identified at the suggestive level (*p* < 5E−06), as listed in Additional file 2, Table S5 These eQTLs were significantly associated with 133 and 671 genes, respectively. The 671 genes were enriched in diverse physiological processes, such as lipid storage, lipoprotein particle binding, and cellular response to lipid (See Additional file 3, Fig. S1a). The chromosomal distribution of these cis-eQTLs is depicted in Fig. 2b. The highest concentration of cis-eQTLs (317) at the genome-wide level was observed on Sus scrofa chromosome (SSC) 15, followed by SSC4 with 243 cis-eQTLs. At the suggestive level, SSC7 and SSC9 had the highest occurrences of 1,291 and 989 cis-eQTLs, respectively (Fig. 2c). Analysis of the number of cis-eQTLs per associated gene revealed that approximately 40% of the 671 genes were influenced by one cis-eQTL and approximately 15% by two. Following an increase in the number of cis-eQTLs per associated gene, the number of these genes gradually declined (Fig. 2d). Notably, some genes were associated with more than 100 cis-eQTLs; for instance, the *PDE7A* gene was significantly associated with 197 cis-eQTLs at the genome-wide level. These findings indicated that gene expression in Laiwu pigs is mainly regulated by multiple genetic loci, and the results are likely due to a higher degree of genome linkage in some eQTL regions of Laiwu pigs. Furthermore, quantile–quantile plot of the tested SNPs is shown in Additional file 3, Fig. S1b confirming the absence of systematic bias in eQTL mapping.

### Integrative analysis of pQTLs affecting IMF content traits detected in a previous GWAS with cis-eQTLs

**Fig. 3:**
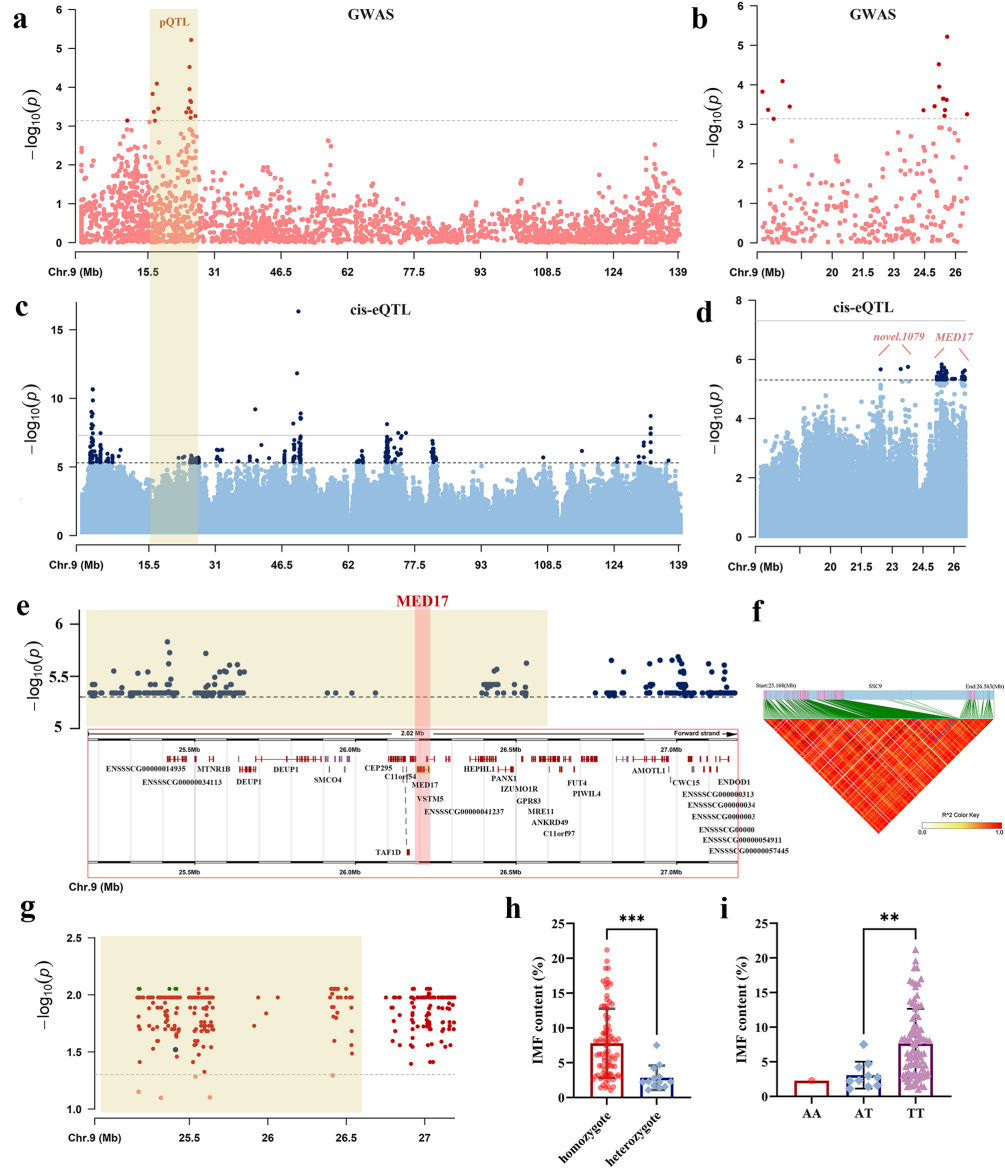
Integrative analysis of intramuscular fat (IMF) content-associated phenotypic quantitative trait loci (pQTL) and cis-expression quantitative trait loci (cis-eQTL) on chromosome 9 (SSC9**)**. **a** Manhattan plot of genome-wide association study (GWAS) for IMF content on SSC9 in Lulai Black pigs, highlighting significant SNPs above the significance threshold (*p* < 6.09E−06). **b** Manhattan plot of GWAS within the pQTL region on SSC9 in Lulai Black pigs. **c** Distribution of cis-eQTLs on SSC9 in Laiwu pigs. **d** Cis-eQTLs within the pQTL region on SSC9, demonstrating four and 228 cis-eQTLs associated with *novel.1079* and *MED17*, respectively. Grey and dotted lines in figure (**c**) and (**d**) marked the significant thresholds for the genome-wide level (*p* < 5E−08) and the suggestive level (*p* < 5E−06), respectively. **e** The overlapped region of IMF content-associated pQTL and cis-eQTLs for *MED17* (*p* < 5E−06). The upper plot depicts significant cis-eQTLs for *MED17*, whereas the bottom details the gene distribution of this genomic region. The red rectangle indicates the location of the *MED17* genes. **f** Linkage disequilibrium (LD) pattern in the overlapped region of pQTL for IMF content and cis-eQTLs for *MED17* expression on SSC9 in Laiwu pigs. The triangles indicate different LD blocks (pairwise SNP R^2^ > 0.90) within this chromosome segment. The blue triangle highlights the LD block containing the most significant SNP for IMF content and the peak cis-eQTL for *MED17* expression. **g** Manhattan plot of the association analysis between significant cis-eQTLs for *MED17* and IMF content. The blue dot highlights the peak cis-eQTL for *MED17*. The green dots highlight 24 completely linked cis-eQTLs for *MED17* showing the strongest association with IMF content. The green-yellow rectangles in figure (**a**), (**c**), (**e**) and (**g**) mark the IMF content-associated pQTL. **h** Box plot of IMF content in individuals with different genotypes of completely linked 24 cis-eQTLs for *MED17*, which showed the strongest association with IMF content. **i** Box plot of IMF content in individuals with different genotypes of the peak cis-eQTL for *MED17*. Box plots show raw, unadjusted IMF content variance between genotypes

In pigQTLdb, 78 pQTLs were detected in Laiwu pigs; however, none were associated with IMF content. In contrast, our previous GWAS of IMF content in Chinese Lulai Black pigs identified the most significant SNP on SSC9, with a *p*-value of 6.09E−06 (Fig. 3a). The most significant pQTL, detected by a 2−LOD drop method, spanned from 16.61 Mb to 26.56 Mb on SSC9 of Sscrofa11.1 reference genome (Fig. 3a, b). The confidence interval of this pQTL overlapped with some of the detected cis-eQTLs (Fig. 3a, c). Within this region, a total of 232 significant cis-eQTLs (*p* < 5E−06) were identified, including four associated with the expression of a newly discovered gene, *novel.1079*, and 228 associated with *MED17* expression (as depicted in Fig. 3b, d and Additional file 2, Table S6). Notably, on SSC9, there was a 2.02 Mb cis-eQTL region (25.17–27.19 Mb), which encompassed 587 cis-eQTLs including the 228 cis-eQTLs mentioned above, and this region was associated with *MED17* expression at the suggestive level (Fig. 3e). These cis-eQTLs were located upstream or downstream of the *MED17* gene and none were within the gene body. Linkage disequilibrium (LD) block analysis from the previous GWAS study highlighted an approximately 2 Mb LD region (24.44–26.56 Mb), which contains the most significant SNP and peak cis-eQTL for *MED17* [8]. This shows high linkage in this region of Lulai Black pigs. Meanwhile, LD analysis with LDBlock software in this study has confirmed the high linkage of this overlapping region in Laiwu pigs. Here, the most significant GWAS SNP and peak cis-eQTL for *MED17* were located within the same LD region (Fig. 3f). Furthermore, an association between 587 cis-eQTLs for *MED17* and IMF content was analyzed using PLINK software. After 100,000 permutation tests, the *p-*values of 582 out of 587 cis-eQTLs were all greater than 0.05 (Fig. 3g). Among these cis-eQTLs, 24 cis-eQTLs in complete linkage exhibited the strongest association with IMF content (*p* = 6.20E-04, Fig. 3g). Notably, individuals with the homozygous genotype of these 24 cis-eQTLs had an IMF content of 7.79% ± 0.53%, which was significantly higher than that of the heterozygous genotype (2.85% ± 0.48%, *p* < 0.001, Fig. 3h). Regarding the peak cis-eQTL for *MED17*, the IMF content of the TT genotype individuals was 7.66% ± 0.81%, significantly higher than that of the AT genotype individuals (3.07% ± 0.56%, *p* < 0.01). There was only one individual with the AA genotype, and its IMF content was 2.29% (Fig. 3i).

### Identification of critical co-expression modules and genes related to IMF content

**Fig. 4:**
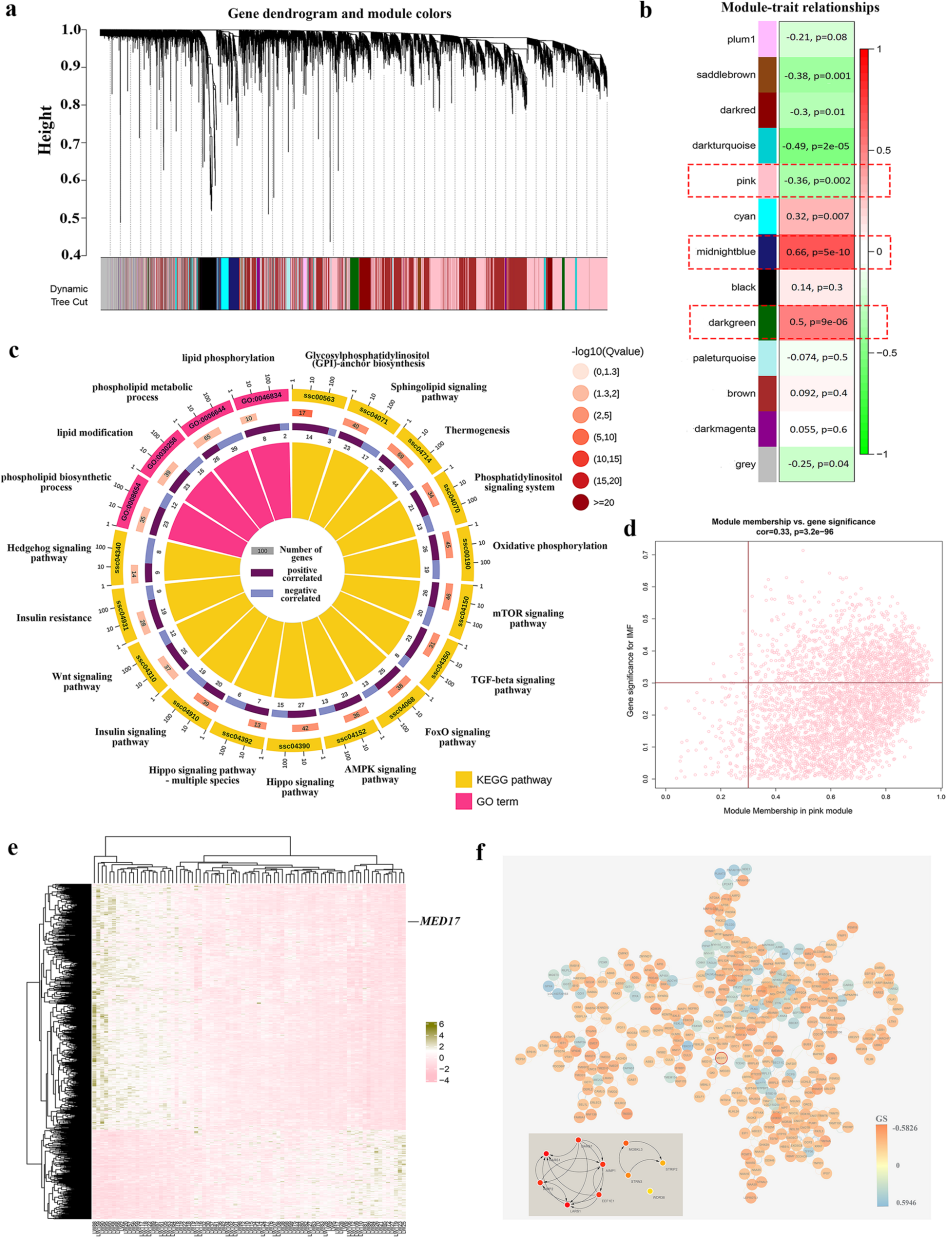
Weighted gene co-expression network analysis (WGCNA) of 70 Laiwu pigs. **a** Cluster dendrogram showing the co-expression modules labeled by colors. **b** Module-trait relationships of each module. The red rectangles highlight three critical co-expression genes related to intramuscular fat (IMF) content, including pink, midnightblue, and darkgreen modules. **c** Some significantly enriched gene ontology (GO) terms and kyoto encyclopedia of genes and genomes (KEGG) pathways (*q* < 0.05) related to IMF content identified with pink module genes. **d** Identification of critical genes in pink module based on *p*-values of gene significance (GS) and module membership (MM). **e** Expression heatmap of critical genes in pink module. **f** Protein–protein interaction (PPI) network for the critical genes in pink module. The lower figure shows the PPI network for the top 10 hub genes in the pink module. The node color indicates GS values

Using the RNA-seq data, we conducted WGCNA on all expressed genes to investigate their correlation with IMF content. Initial sample clustering identified nine individuals who deviated from the rest at a predetermined cutoff height. These individuals were deemed outliers and were excluded from subsequent analysis. WGCNA revealed 13 co-expression modules, with each module containing between 80 and 3760 genes (Fig. 4a). Based on the criteria of MTRs with *p*-values of < 0.01, six modules were found to be significantly correlated with IMF content (Fig. 4b). These modules were: saddlebrown (r = − 0.38, *p* = 1E−04), pink (r = − 0.36, *p* = 2E−03), darkturquoise (r = − 0.49, *p* = 2E−05), cyan (r = 0.32, *p* = 7E−03), midnightblue (r = 0.66, *p* = 5E−10), and darkgreen (r = 0.50, *p* = 9E−06). Detailed gene information for these six IMF-related modules is provided in Additional file 2, Table S7.

Following the identification of the IMF-related co-expression modules via WGCNA, functional enrichment analyses were conducted on the genes within the six IMF-related modules to elucidate the functions associated with fat deposition. Three modules (pink, midnightblue, and darkgreen) showed significant enrichment in GO terms and KEGG pathways related to IMF content (Fig. 4b). These significantly enriched GO terms and pathways (*q* < 0.05) are detailed in Additional file 2, Tables S8 and S9. The pink module’s genes were enriched in 94 pathways and 137 GO terms. Some significantly enriched pathways, such as Sphingolipid signaling pathway (*q* = 3.40E−05) [12, 13], FoxO signaling pathway (*q* = 1.37E−03) [14], AMPK signaling pathway (*q* = 1.89E−03) [15], and Insulin signaling pathway (*q* = 5.54E−03) [16, 17], are all integral to fat deposition (Fig. 4c). Several GO terms associated with lipid modification and metabolism were also significantly enriched, including phospholipid biosynthetic process (*q* = 1.36E−02), lipid modification (*q* = 2.59E−02), phospholipid metabolic process (*q* = 3.20E−02), and protein lipidation (*q* = 4.27E−02) (Fig. 4c). Therefore, the pink was deemed to be one of the critical co-expression modules related to IMF content. The functions of the other IMF-related co-expression modules were presented in Additional file 1, Text S10, as well as in Additional file 2, Table S8 and Table S9.

**Fig. 5:**
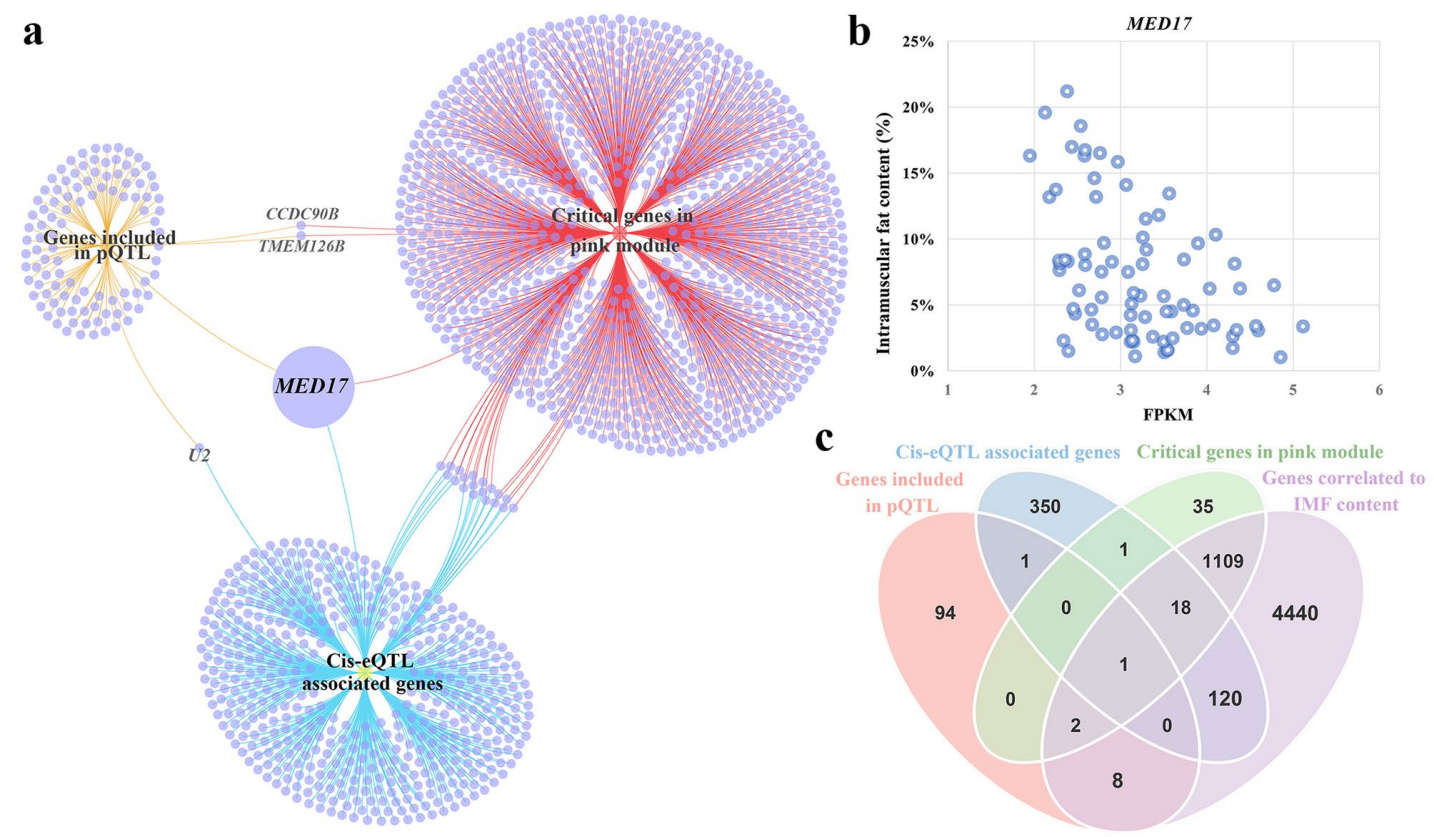
Identification of *MED17* as a candidate gene affecting intramuscular fat (IMF) content based on genome and transcriptome data in pigs. **a** Integrating the results of phenotypic quantitative trait loci (pQTL) mapping, cis-expression quantitative trait loci (cis-eQTL), and Weighted gene co-expression network analysis (WGCNA) identifies *MED17*. **b** The scatter diagram between the Fragments Per Kilobase of exon model per Million mapped fragments (FPKM) value of *MED17* and IMF content. **c** Venn diagram of genes identified by pQTL mapping, cis-eQTL mapping, WGCNA, and correlation analysis

In addition, we further identified the critical genes within the pink module. Employing criteria of both GS and MM *p*-values under 0.01, 1,166 out of 3,760 genes were identified as critical genes (as depicted in Fig. 4d and Additional file 2, Table S10). The expression of these genes varied in different individuals (Fig. 4e). Notably, *MED17*, which was identified as one of the genes associated with IMF content through the integrated analysis of cis-eQTL and IMF-related pQTL, emerged as a critical gene in the pink module. According to the Maximal Clique Centrality algorithm, 10 of the critical genes (*RARS1*, *DARS1*, *AIMP1*, *EEF1E1*, *LARS1*, *AIMP2*, *MOBKL3*, *STRN3*, *STRIP2*, and *WDR36*) were highlighted hub genes because of their encoding proteins with high stability and scores in the PPI network (Fig. 4f). Although *MED17* was not included in the hub genes, its encoding protein interacted with four proteins (GTF2B, QKI, MED23, and MED13) of this module (Fig. 4f). Furthermore, we investigated whether the genes within the module are regulated by cis-eQTLs. In addition to *MED17*, 22 other genes in the pink module, such as *TXNDC15*, *TMEM19*, *PDE9A*, and *RCOR3*, were identified as being associated with cis-eQTLs (See Additional file 2, Table S10). Hence, integrating the results of QTL mapping, cis-eQTL, and WGCNA, *MED17* was considered as a potential candidate gene of IMF content (Fig. 5a).

### Genes correlated to IMF content

To further analyze the relationship between the *MED17* gene and the IMF content values, correlation analysis was performed. The log_2_-transformed gene expression levels and phenotypic values of IMF content were pre-adjusted using a fixed linear model. Among the 14,588 expressed genes, *MED17* was significantly negatively correlated with IMF (FDR = 1.58E−02, Fig. 5b). Additionally, apart from *MED17*, another 181 other genes, that were significantly correlated with IMF content, such as *PTGR1*, *BOK*, and *ENSSSCG00000051428*, were cis-eQTL-associated genes (See Additional file 2, Table S11). Table 1 shows the top 10 IMF content-related genes affected by cis-eQTLs. Finally, integrating the results of QTL mapping, cis-eQTL, WGCNA, and correlation analysis, *MED17* was identified as a pivotal candidate gene affecting IMF content in pigs (Fig. 5c). Overall, through an integrative analysis that combined cis-eQTL mapping, pQTL mapping from previous GWAS data, WGCNA, and correlation analysis, we identified *MED17* as a novel candidate gene involved in the regulation of IMF content in pigs.

**Table 1:** Top 10 genes significantly related to intramuscular fat content (*q* ≤ 0.05) and affected by cis-expression quantitative trait loci (cis-eQTLs) in Laiwu pigs

Gene Name	Location	Coefficient	FDR	Number of cis-eQTLs	Gene description
*PTGR1*	1:252373279-252395948	0.68	4.70E−09	6	Prostaglandin reductase 1
*BOK*	15:140171143-140184124	0.67	1.97E−08	1	BCL2 family apoptosis regulator BOK
*ENSSSCG00000051428*	10:65559698-65572893	0.61	1.00E−06	1	–
*APCDD1*	6:97992228-98026768	0.61	1.00E−06	3	APC down-regulated 1
*CSAD*	5:18379340-18410212	0.59	2.64E−06	1	Cysteine sulfinic acid decarboxylase
*SFRP1*	17:10436426-10489748	0.58	4.05E−06	5	Secreted frizzled related protein 1
*OPRL1*	17:62897727-62913754	0.57	6.41E−06	1	Opioid related nociceptin receptor 1
*TXNDC15*	2:137060893-137080107	− 0.56	1.28E−05	68	Thioredoxin domain containing 15
*PRKACB*	6:129519962-129635517	− 0.53	3.32E−05	9	Protein kinase cAMP-activated catalytic subunit beta
*NAB2*	5:22400277-22406419	0.50	9.96E−05	1	NGFI-A binding protein 2

### *MED17* affects lipid deposition in 3T3-L1 cells

**Fig. 6:**
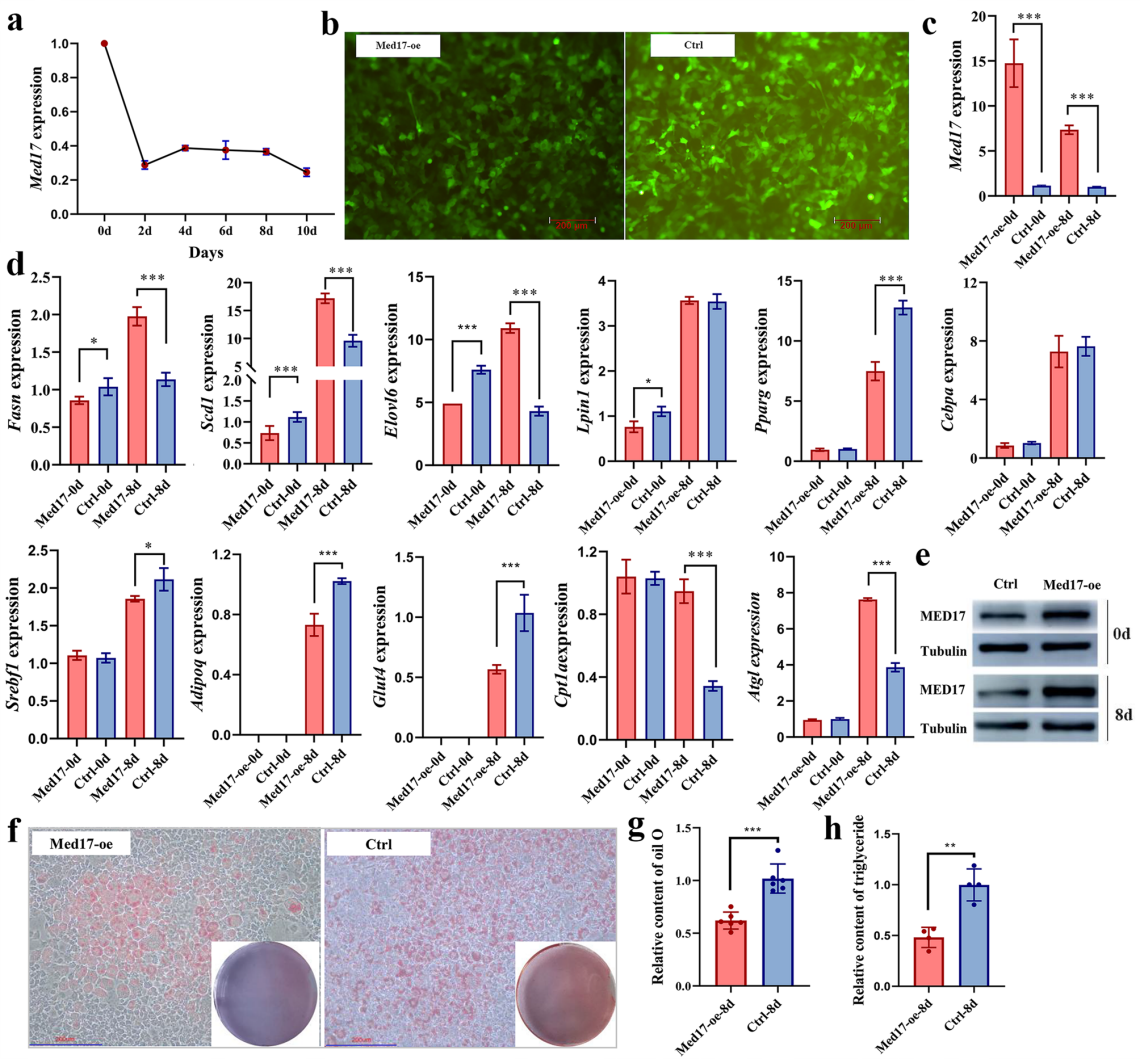
Effect of adenovirus-mediated overexpression of mouse *Med17* on the differentiation of 3T3-L1 cells into adipocytes. **a** The *Med17* mRNA level during 3T3-L1 differentiation. **b** Adenovirus transfection efficiency in 3T3-L1 cells after transfected for 48 h. Scale bar, 200 μm. **c** The mRNA level of *Med17* in the 3T3-L1 cells at two days post-infection with *Med17*-expressing adenovirus and at day eight of adipocyte differentiation. **d** The mRNA levels of some critical genes involved in adipogenesis in the 3T3-L1 cells at two days post-infection with *Med17*-expressing adenovirus and at day eight of adipocyte differentiation. **e** Med17 Protein levels in the 3T3-L1 cells at two days post-infection with *Med17*-expressing adenovirus and at day 8 of adipocyte differentiation. **f** Oil Red O staining of 3T3-L1 cells at day eight of differentiation. The upper figures display stained cells observed under a microscope, with a scale bar indicating 200 μm. The right lower images illustrate the stained status of cells in an entire well of a six-well plate for the *Med17* overexpression group and the control group, respectively. **g** Relative content of Oil Red O in 3T3-L1 cells detected at 490 nm after being eluted with 100% isopropanol. **h** Relative triglyceride content in 3T3-L1 cells at day eight of differentiation. Data in (**c**, **d**, **g**, and **h**) are presented as mean ± standard error (SE) from three to four independent experiments. *, *p* < 0.05; **, *p* < 0.01; ***, *p* < 0.001. Med17-oe represents the 3T3-L1 cell group transfected with adenovirus expressing the mouse *Med17* gene. Ctrl represents the negative control

3T3-L1 murine preadipocyte cell line was used to determine the regulatory role of *MED17* in fat deposition. The mouse *Med17* mRNA levels decreased immediately after adipogenic differentiation and remained low throughout the differentiation process in 3T3-L1 cells (Fig. 6a). Furthermore, gain-of-function experiments were conducted via 2-day transfection with the adenovirus carrying the mouse *Med17* gene (Fig. 6b). The *Med17* mRNA level of 3T3-L1 cells significantly increased 12.9-fold and 7.3-fold respectively at days 0 and 8 of differentiation compared with that in the control group (Ctrl), along with corresponding increases in MED17 protein levels (Fig. 6c, e). Meanwhile, after 2 days post-infection, *Med17* overexpression (Med17-oe) led to the significant downregulation of adipogenesis-related genes, including *Fasn*, *Scd1*, *Elovl6*, and *Lpin1* (*p* < 0.05, Fig. 6e). Although the expression of *Pparg* and *Cebpa*, two critical transcription factors involved in adipogenesis, was reduced after *Med17* overexpression, these changes were not statistically significant (*p* > 0.05, Fig. 6e). At eight days of adipogenic differentiation, the mRNA levels of *Pparg* and some other adipogenesis-related genes, including *Srebf1*, *Adipoq*, and *Glut4*, were significantly decreased, whereas the levels of *Cpt1a* and *Atgl—*regulating fatty acid oxidation and triglyceride hydrolysis respectively—were significantly increased (*p* < 0.05, Fig. 6d). Surprisingly, at eight days, the expression of *Fasn*, *Scd1*, and *Elovl6*, which are all involved in fatty acids synthesis, was significantly upregulated (*p* < 0.05, Fig. 6d). Oil Red O staining of 3T3-L1 cells after eight days of adipogenic differentiation revealed a reduction in intracellular lipid droplets in cells overexpressing *Med17* compared with that in the control group, which exhibited abundant lipid droplets (Fig. 6f, g). Furthermore, *Med17* overexpression adipocytes accumulated lower TG levels (Fig. 6h). These results strongly support the role of *MED17* in the regulation of adipogenesis and suggest that it is a potentially critical gene for pig IMF content.

## Discussion

### Integrative analysis of eQTL and pQTL to obtain insight into IMF content trait in pigs

In the field of animal science and the meat industry, research on IMF in pigs is of paramount importance. IMF content is a crucial determinant of pork quality, directly influencing sensory attributes such as tenderness, juiciness, and flavor. This not only meets consumers’ demands for high-quality meat products but also serves as a key factor for meat processing enterprises to enhance their product competitiveness. To better understand the genetic basis of IMF content and develop effective breeding strategies, GWAS studies are commonly employed to identify SNPs associated with IMF content in pigs, and many have indicated that some of the SNPs associated with complex traits may also be eQTLs [18]. By integrating eQTL results with GWAS findings, the analytical power of GWAS for complex trait analyses can be remarkably enhanced [19, 20]. In this study, we applied this integrated approach to identify candidate genes for IMF content in pigs.

We integrated RNA-seq and whole-genome resequencing data to identify cis-eQTLs that affect IMF content in 79 Laiwu pigs. At the genome-wide level (*p* < 5E−08), 1,337 cis-eQTLs were detected, and at the suggestive level (*p* < 5E−06), 9,763 cis-eQTLs were detected (Fig. 2b, c). In farm animals, SNPs utilized for eQTL mapping are typically derived from RNA-seq data or SNP chips [4, 21, 22]. In numerous studies, the FDR adjustment has been applied, with eQTL significance thresholds set at 0.05 or 0.01 [21, 23]. In the present study, we used SNPs identified in whole-genome DNA sequences at an approximate depth of ten-fold for eQTL mapping. Hence, we aligned our criteria for cis-eQTL detection with the significance thresholds employed in GWAS studies based on whole-genome variations in Chinese indigenous pigs [24]. This approach was also adopted by Zhang et al. [25], who identified eQTLs in pigs using whole-genome variants and analogous criteria for cis-eQTL detection. In this study, 79 Laiwu pigs were used for eQTL mapping. Despite relatively small sample sizes (< 100 individuals), meaningful results have been reported [4, 26]. In addition to factors, such as sequencing coverage depth, significance threshold, and sample size, other elements, like gene expression measurement techniques, breeds studied, and statistical models, can also influence the number of detected cis-eQTLs. Despite these variables, the number of cis-eQTLs identified in the present study was within the expected ranges. For example, Liu et al. [27] detected 7,192 cis-eQTLs (*p* ≤ 1.33E-03, FDR ≤ 0.05) using Illumina porcine 50 K genotyping and RNA-seq data from 189 Duroc × Luchuan crossbred pigs. This consistency supports the validity of our approach and the results.

The combination of eQTL and QTL analysis has proven to be an effective method for revealing the genetic architecture of complex traits in farm animals, particularly in terms of meat quality [5, 28, 29], production traits [30], fat content [31], and residual feed intake [4]. In this study, by leveraging the outcomes of eQTL analysis, we focused on identifying candidate genes affecting the IMF content trait by integrating the QTL mapping results. Laiwu pigs are distinguished from other breeds by their remarkably high IMF content. This unique trait is closely linked to specific gene expression profiles, which differ markedly from those of other breeds, particularly with respect to IMF content [32, 33]. Given the breed-specific nature of eQTL discovery, we utilized Laiwu pigs and their crossbreed, Lulai Black pigs, which possess approximately 65% Laiwu ancestry, for QTL and cis-eQTL colocalization. Although no QTLs specific to Laiwu and Lulai Black pigs were found in pigQTLdb, our previous GWAS study identified significant SNPs associated with IMF content in Lulai Black pigs [8]. By aligning these pQTLs with eQTLs, we identified co-localized cis-eQTLs associated with the expression of *novel.1079* and *MED17* (Fig. 3a–d). Colocalization refers not only to exact overlaps between QTL and eQTL regions but also to determining whether a single variant is responsible for both GWAS and eQTL signals in a locus, thereby pinpointing potential causal variants [4]. In this study, we observed a shared region between the pQTL and cis-eQTL regions associated with *MED17*, which exhibited high linkage in both laiwu and Lulai Black pig populations (Fig. 3f). These results indicated that *MED17* is a potential candidate causal gene for IMF content in pigs. Similarly, Liu et al. [5] identified candidate genes, like *GALNT15* and *HTATIP2*, which influence meat quality traits by colocalizing the GWAS signal with peak cis-eQTLs. These findings demonstrate that integrating QTL and eQTL analyses is a powerful approach for identifying candidate genes for complex traits in farm animals.

### *MED17* as a critical gene in one critical module related to IMF-content

Based on the RNA-seq data, we used WGCNA to explore the relationships between IMF content and gene expression profiles. We previously conducted a similar analysis using transcriptome data from Duroc pigs, identifying several genes related to lipid formation, such as *ADIPOQ*, *PPARG*, *CIDEA*, and *FABP4*, which were also closely related to IMF content [34]. In this study, we identified six modules that were significantly correlated with IMF content (MTR* p* < 0.01, Fig. 4b). Functional enrichment analysis of the co-expressed genes was performed to further explore the function of these modules in relation to IMF content. Of these, three modules were deemed to be critical co-expression modules related to IMF content (Fig. 4b). The pink module genes were significantly enriched in IMF-related pathways, such as the FoxO, AMPK, and insulin signaling pathway (Fig. 4c). All these pathways play critical roles in regulating glucose and lipid metabolism. In muscle and fat cells, the clearance of circulating glucose depends on the insulin-stimulated translocation of the GLUT4 glucose transporter to the cell surface. Insulin also has a profound effect on lipid metabolism by increasing lipid synthesis in the liver and fat cells and attenuating fatty acid release from triglycerides [35, 36]. FoxO proteins in the FoxO signaling pathway, particularly FoxO1, play a vital role in regulating whole-body energy metabolism [37] and mediating the inhibitory action of *IGF-1* on key functions in glucose and lipid metabolism [14, 38]. *AMPK* is the central gene of the AMPK signaling pathway. It mediates hormonal signaling that regulates the effects of hormones related to glucose and lipid metabolism, such as leptin, ghrelin, adiponectin, glucocorticoids and insulin [39]. Moreover, Yao et al. [15] discovered that the AMPK signaling pathway is associated with IMF content in pigs. In addition, pink module genes were also enriched in GO terms related to lipid modification and metabolism, including phospholipid biosynthetic process, lipid modification, and phospholipid metabolic process (Fig. 4c). As the function of the pink module genes, we considered the pink module as one of the critical modules related to IMF content. Within this module, we identified 1,166 critical genes affecting IMF content out of 3,760 module genes, with *MED17* emerging as a critical gene. The expression of *MED17* was significantly correlated with IMF content (FDR = 1.58E−02). By integrating QTL mapping, cis-eQTL analysis, WGCNA, and correlation analysis, we considered *MED17* to be an important candidate gene affecting IMF content. Our strategy for identifying candidate genes was similar to that of Meng et al. [40], who identified novel causal genes affecting low bone mineral density in humans by integrating GWAS, eQTL, and WGCNA. Similarly, Liu et al. [5] integrated GWAS loci, eQTL, WGCNA, and QTT to screen for candidate genes influencing meat quality traits in pigs.

### Vital role of *MED17* in fat deposition

In this study, we identified *MED17* as an important candidate gene that affects IMF content. MED17 is one subunit of the head module of the Mediator complex in mammals and serves as a general target of transcription factor (TF) activation domains by communicating regulatory signals from DNA-bound TFs directly to RNA polymerase II [41–43]. Different subunits of the intermediary complex can specifically interact with various TFs involved in the regulation of metabolic genes. Several Mediator subunits, such as MED1 [44, 45], MED14 [46], and MED23 [47], have been shown to play roles in adipogenesis and lipogenic gene expression. The role of *MED17* in adipogenesis has been primarily explored in the liver, where it regulates lipogenic activity through the liver X receptor [48]. Additionally, Viscarra et al. [49] found that the CK2-mediated phosphorylation of MED17 at Ser53 in the liver is essential for transcriptional activation of adipogenic genes in response to insulin.

The biological function of *MED17* in regulation of lipid deposition in the muscle and fat cells has rarely been studied. In this study, PPI analysis revealed that MED17 interacts with GTF2B, QKI, MED23, and MED13 within the critical IMF-related module. These interacting proteins with MED17 were involved in energy metabolism, adipogenesis and lipid homeostasis. Specifically, the RNA-binding protein QKI plays an important role in restricting energy consumption of adipose tissue by regulating *UCP1* and *PGC1α*, thereby promoting high-fat diet-induced obesity, while *QKI* deficiency enhances brown fat thermogenesis and white fat browning [50]. Mediator subunit MED23 has been demonstrated to serve as a critical mediator bridging insulin signaling with the adipogenesis transcriptional cascade [47]. MED23 deficiency promotes smooth muscle cell differentiation while inhibiting adipocyte differentiation in multipotent mesenchymal stem cells [51]. Additionally, cardiac-specific overexpression of MED13 in transgenic mice leads to a lean phenotype via enhanced lipid uptake, β-oxidation, and mitochondrial biogenesis in white adipose tissue and liver [52].

In pigs, the expression of *MED17* is negatively correlated with IMF content (Fig. 5b). During the early stages of adipocyte differentiation, *MED17* mRNA expression notably decreased and remained low in developing 3T3-L1 adipocytes (Fig. 6a), suggesting that lower *MED17* expression levels may promote fat deposition. In yeast, *MED17* depletion impairs preinitiation complex assembly, resulting in global transcriptional downregulation [53]. Regarding many basic functions of the Mediator complex conserved between yeasts and humans [54], in order to study the role of *Med17* in adipogenesis and avoid transcriptional dysregulation, we overexpressed *Med17* expression in 3T3-L1 cells instead of interfering with its expression. Although *Med17* overexpression did not significantly decrease the expression of *Pparg* and *Cebpa*, which are key regulatory factors of adipogenic differentiation, in undifferentiated 3T3-L1 cells [55], *Pparg* expression was significantly reduced in differentiated adipocytes (Fig. 6d). This further supports the hypothesis that low *MED17* expression is conducive to adipogenesis. We also observed significant changes in the expression of genes related to lipogenesis, fatty acid metabolism, and glucose metabolism, including *Srebf1* [56], *Adipoq* [57], *Glut4* [58], *Atgl* [59], and *Cpt1a* [60] (Fig. 6d). These findings were consistent with the results from Oil Red O staining and the TG assay (Fig. 6f–h).

Notably, fatty acid synthesis-related genes (*Fasn*, *Scd1*, and *Elovl6*) were downregulated following *Med17* overexpression, whereas these genes were significantly upregulated in adipocytes after the induction of 3T3-L1 differentiation (Fig. 6d). This may result from a compensatory response, in which cells attempt to compensate for the decline in other lipid metabolic pathways by increasing fatty acid synthesis. This aligns with prior studies: Kotani et al. [61] found that the tissues of mice with *Glut4* glucose transporter deficiency adapted by increasing the utilization of fatty acids; similarly, transgenic mice with liver-specific AMPK-α1 activation exhibit compensatory upregulation of cholesterol and fatty acid synthesis genes [62]. Moreover, mice with conditional liver SCAP deficiency display increased adipose tissue fatty acid synthesis as a compensatory response [63]. The compensatory response hypothesis should be investigated after confirming whether *FASN*, *SCD1*, or *ELOVL6* serve as direct transcriptional targets of *MED17*.

These cellular data indicate that *MED17* might regulate fat deposition at multiple levels, thereby influencing both preadipocyte differentiation and adipocyte metabolic status. The detailed regulatory mechanisms of *MED17* will be explored in future studies.

## Conclusion

Overall, through an integrative analysis that combined cis-eQTL mapping, pQTL mapping from previous GWAS data, WGCNA, and correlation analysis, we identified *MED17* as a novel candidate gene involved in the regulation of IMF content in pigs. Functional validation in 3T3-L1 preadipocytes demonstrated that *Med17* overexpression significantly reduced adipogenic differentiation, accompanied by the altered expression of some critical adipogenesis-related genes. These results provide valuable insights into the genetic architecture of IMF deposition, with *MED17* identified as a critical candidate gene. Moreover, this study lays the foundation for improving pork quality through increasing IMF content.

## Supplementary Information

Below is the link to the electronic supplementary material.

Supplementary Material 1.

Supplementary Material 2.

Supplementary Material 3.

## Data Availability

The datasets supporting the conclusions of this article is in the Genome Sequence Archive in National Genomics Data Center, China National Center for Bioinformation/Beijing Institute of Genomics, Chinese Academy of Sciences (GSA: CRA019689, CRA019814) that are publicly accessible at https://ngdc.cncb.ac.cn/gsa.
