# Supplementary material for: Integrative analysis of genome and transcriptome reveals a novel regulator for pork intramuscular fat content
Source: Genet Sel Evol. 2025 Nov 6;57:67. doi: 10.1186/s12711-025-01014-9 (PMC12590886; doi:10.1186/s12711-025-01014-9)
Supplement: Supplementary file 1 — Supplementary Material 1. [file 12711_2025_1014_MOESM1_ESM.docx]

Text S1 Genomic DNA genotyping

Extracted genomic DNA was analyzed for quality and quantity using a Nanodrop 2000 spectrophotometer (Thermo Fisher Scientific, Waltham, MA, USA) and a Qubit Fluorometer (Thermo Fisher Scientific). Genotyping was performed using 350 bp paired-end libraries constructed by TruSeq Library Construction Kit (Novogene, Beijing, China) on an Illumina HiSeq instrument. The sequence data for each individual reached more than 10-fold depth. After filtering the raw reads and removing the low-quality reads, clean reads were obtained and mapped to the Sscrofa11.1 reference genome using BWA software (version 0.7.15). SNPs per sample were called, and duplicates were removed using SAMTOOLS [1].

Text S2 RNA sequencing

RNA integrity was assessed using an RNA Nano 6000 Assay Kit on a Bioanalyzer 2100 system (Agilent Technologies, Santa Clara, CA, USA). The mRNA was subsequently isolated using poly-T oligo-attached magnetic beads and fragmented using divalent cations. Libraries for sequencing were prepared using the NEBNext® Ultra™ RNA Library Prep Kit for Illumina (Ipswich, MA, USA) and were quantified on the Agilent Bioanalyzer 2100 system. Paired-end sequencing was conducted on an Illumina NovaSeq platform, producing approximately 150 bp-sized reads. Clean data were obtained by filtering low-quality reads and those containing adapters and N bases. Paired-end clean reads were aligned to the Sscrofa11.1 reference genome using Hisat2 v2.0.5, and the gene read numbers were determined using featureCounts v1.5.0-p3. Subsequently, fragments per kilobase of transcript sequence per millions base pairs sequenced (FPKM) was calculated based on gene length and read counts mapped to each gene. Genes exhibiting an FPKM value greater than 0.1 in over half of the samples were considered expressed and were included in further analyses [2].

Text S3 Weighted gene co-expression network analysis (WGCNA)

Initially, hierarchical clustering of all samples was performed using R function “clust” with a cutoff height of 15000 to identify outlier individuals. Nine pigs appeared to be markedly deviant and were subsequently removed from the dataset. To construct an unsigned co-expression network, an adjacency matrix was generated using Pearson correlation coefficients between each pair of genes under a soft-power threshold power (*β*) of 11, achieving a scale-free topology index (R^2^) of 0.85. Next, to enhance network robustness against spurious connections and missing connections due to random noise, the adjacency matrix was transformed into a topology overlap matrix (TOM). The corresponding dissimilarity TOM (calculated as 1−TOM) served as the distance metric for the hierarchical clustering of genes. Genes exhibiting similar expression patterns were clustered into the same modules and assigned colors based on hierarchical average linkage clustering using a dynamic tree-cutting algorithm with the default parameters of minModuleSize set at 30 and mergeCutHeight at 0.25. For each co-expression module, the first principal component of the gene expression matrix, termed the module eigengene (ME), represented the collective expression profile of the module. Pearson correlations between MEs and IMF contents were calculated to estimate module-trait relationships (MTR). Within the modules, gene significance (GS) and module membership (MM) were assessed using Pearson correlations between the expression profiles and both IMF content and MEs, respectively.

Text S4 Protein-protein interaction (PPI) network analysis

PPI network for these critical genes was generated using STRING 12.0 (http://string-db.org/), with a confidence inter-action score of 0.7 (high confidence). The network was constructed using various active interaction sources, including gene neighborhood, gene fusion, gene co-occurrence, text mining, co-expression, protein homology, experimentally determined, and curated databases as active interaction sources. Cytoscape software version 3.9.1 was used for PPI network visualization, and the Cytoscape cytoHubba plugin was used to further filter and obtain the top 10 hub genes.

Text S5 Adenoviral plasmid construction

The cDNA of the mouse *Med17* gene (NC_000075.7) was amplified using reverse transcription polymerase chain reaction (PCR) and subcloned into an adenovirus plasmid (pADM-FH-GFP, WZ Biosciences, Jinan, China). This adenoviral plasmid, which was not subcloned with any exogenous genes, served as a negative control. Both the recombinant adenoviral vectors expressing *Med17* and the negative control vector were propagated in HEK293 cells. After that, the adenoviruses were harvested, purified, and their titers were quantified using the gradient dilution method.

Text S6 3T3-L1 cell culture and adipocyte differentiation

3T3-L1 cells were cultured in high glucose Dulbecco’s modified Eagle’s medium (DMEM, Gibco, Grand Island, NY, USA) supplemented with 10% newborn bovine serum (Gibco) and 100 IU/mL penicillin-streptomycin (Gibco), and were maintained at 37°C in a humidified atmosphere with 5% CO_2_. After adenovirus transfection, the cells were incubated for 48 h in growth medium containing DMEM supplemented with 10% fetal bovine serum (FBS, Gibco). Upon reaching 90% confluence, differentiation into adipocytes was initiated by culturing in differentiation medium comprising DMEM supplemented with 10% FBS, 1 μM dexamethasone (Sigma-Aldrich, Saint Louis, Mo, USA), 0.5 mM IBMX (Sigma-Aldrich), and 5 μg/mL insulin (Sigma-Aldrich). After four days, the culture was sustained in maintenance medium containing DMEM, 10% FBS, and 10 μg/mL insulin for another four days.

Text S7 Quantitative real-time PCR (qRT-PCR) analysis

Total RNA was extracted from 3T3-L1 cells which were infected with adenovirus for 48 h and were subsequently differentiated for eight days, using TRNzol Universal reagent (TIANGEN, Beijing, China) according to the manufacturer’s instructions. Reverse transcription of 800 ng of total RNA to cDNA was performed using the PrimeScript™ RT reagent Kit with gDNA Eraser (TaKaRa). The qRT-PCR was conducted using TB Green® Premix Ex Taq™ II FAST qPCR (TaKaRa) on a Roche LightCycler 480 System. The relative expression levels of *Med17* and some genes associated with adipogenesis were quantified using the 2^−ΔΔCT^ method. Gene specific primers were used for each target gene, with *Tbp* serving as the internal control (See Additional file 2, Table S1).

Text S8 Protein extraction and western blot assay

Total protein was isolated from 3T3-L1 cells infected with adenovirus for 48 h and differentiated for eight days, using high-strength RIPA lysis buffer (Servicebio, Wuhan, China). The protein concentration was determined using a BCA protein assay kit (Beyotime, Shanghai, China). Proteins were separated and analyzed by western blotting as described in our previous study [3]. Primary antibody against MED17 (Proteintech Group, Rosemont, Illinois, USA) was applied at 1:800 dilution. Horseradish peroxidase-conjugated goat anti-rabbit IgG (Proteintech Group) was utilized as the secondary antibody at a dilution of 1:2,500. Chemiluminescence was detected via a Tanon-5200 Chemiluminescent Imaging System (Tanon Science & Technology, Shanghai, China).

Text S9 Lipid content assessment in 3T3-L1 cells

Lipid accumulation in 3T3-L1 cells was evaluated on day eight of adipogenic differentiation using the Oil Red O Staining kit (Solarbio, Beijing, China). After staining, Oil Red O dye was extracted from the stained adipocytes using 100% isopropyl alcohol for 10 min. The concentration of the eluted dye was quantified by measuring the absorbance at 490 nm. Furthermore, the cells on day eight of differentiation were also lysed using 1% Triton X-100 to assess the total triglyceride (TG) content. The lysate was analyzed following the protocol of the TG assay kit provided by Nanjing Jiancheng Bioengineering Institute (China).

Text S10 Functional enrichment results of other intramuscular fat-related co-expression modules except the pink module

For the midnightblue module, genes within it were signiﬁcantly enriched in 13 pathways and 137 GO terms (*q* < 0.05), as documented in Additional file 2, Table S8 and Table S9. Approximately half of these pathways were directly related to lipid deposition, such as the PPAR signaling pathway (*q* = 5.93E−04), fatty acid metabolism (*q* = 1.35E−02), and the regulation of lipolysis in adipocytes (*q* = 4.17E−02). Some significantly enriched GO terms were related to lipid regulation and fatty acid metabolism, such as lipid homeostasis (*q* = 2.81E−02), positive regulation of fat cell differentiation (*q* = 3.89E−02), lipid biosynthetic process (*q* = 3.89E−02), and lipid droplet (*q* = 4.34E−02). In contrast, the darkgreen module was significantly enriched in 14 pathways and 16 GO terms (*q* < 0.05). We found some of these pathways and GO terms to be related to glycometabolism and carbohydrate metabolism, such as glycolysis/gluconeogenesis (*q* = 1.86E−02), galactose metabolism (*q* = 2.68E−02), glucose 6-phosphate metabolic process (*q* = 3.25E−02), carbon metabolism (*q* = 1.59E−02), and carbohydrate binding (*q* = 1.47E−02). Glycometabolism and carbohydrate metabolism influence lipid synthesis and deposition by modulating insulin release, converting it directly to fatty acids, and activating specific metabolic pathways [4-6], thereby identifying the darkgreen module as being related to IMF content. For the saddlebrown and darkturquoise modules, the module genes were involved in multiple processes, such as histone modification, chromatin organization and modification, and mRNA metabolism. However, they were not enriched in IMF-related GO terms and pathways (See Additional file 2, Table S12). Consequently, considering both module MTR and module gene function, midnightblue and darkgreen modules were also deemed to be critical co-expression modules related to IMF content. In the midnight module, 190 out of 302 genes, and in the darkgreen module, 221 out of 299 genes were identified as critical genes, respectively (See Additional file 2, Table S10, Additional file 3, Figure S2a and S2b). The PPI network for these critical genes was shown in Additional file 3, Figure S2c and S2d.

Reference

1. Li H, Handsaker B, Wysoker A, Fennell T, Ruan J, Homer N, et al. The sequence alignment/map format and SAMtools. Bioinformatics. 2009;25:2078-9.

2. Zhang Y, Sun Y, Wu Z, Xiong X, Zhang J, Ma J, et al. Subcutaneous and intramuscular fat transcriptomes show large differences in network organization and associations with adipose traits in pigs. Sci China Life. 2021;64:1732-46.

3. Zhao X, Jia W, Wang J, Wang S, Zheng Q, Shan T. Identification of a candidate gene regulating intramuscular fat content in pigs through the integrative analysis of transcriptomics and proteomics data. Journal of Agricultural and Food Chemistry. 2023;71:19154-64.

4. Wang X, Liu J, Yang Y, Zhang X. An update on the potential role of advanced glycation end products in glycolipid metabolism. Life Sci. 2020;245:117344.

5. Xue LL, Chen HH, Jiang JG. Implications of glycerol metabolism for lipid production. Prog Lipid Res. 201712.

6. Hardie DG. Organismal carbohydrate and lipid homeostasis. Cold Spring Harb Perspect Biol. 2012;4:a006031.
