# Supplementary material for: Integrative analysis of genome and transcriptome reveals a novel regulator for pork intramuscular fat content
Source: Genet Sel Evol. 2025 Nov 6;57:67. doi: 10.1186/s12711-025-01014-9 (PMC12590886; doi:10.1186/s12711-025-01014-9)

Figure S1 Functional enrichment analysis of expression quantitative trait loci (eQTL) associated genes and quantile-quantile plot analysis for eQTL.

(a) Top 10 Gene ontology (GO) terms of eQTL associated genes. (b) Quantile-quantile plot of −log10(*p*-value) of eQTL.


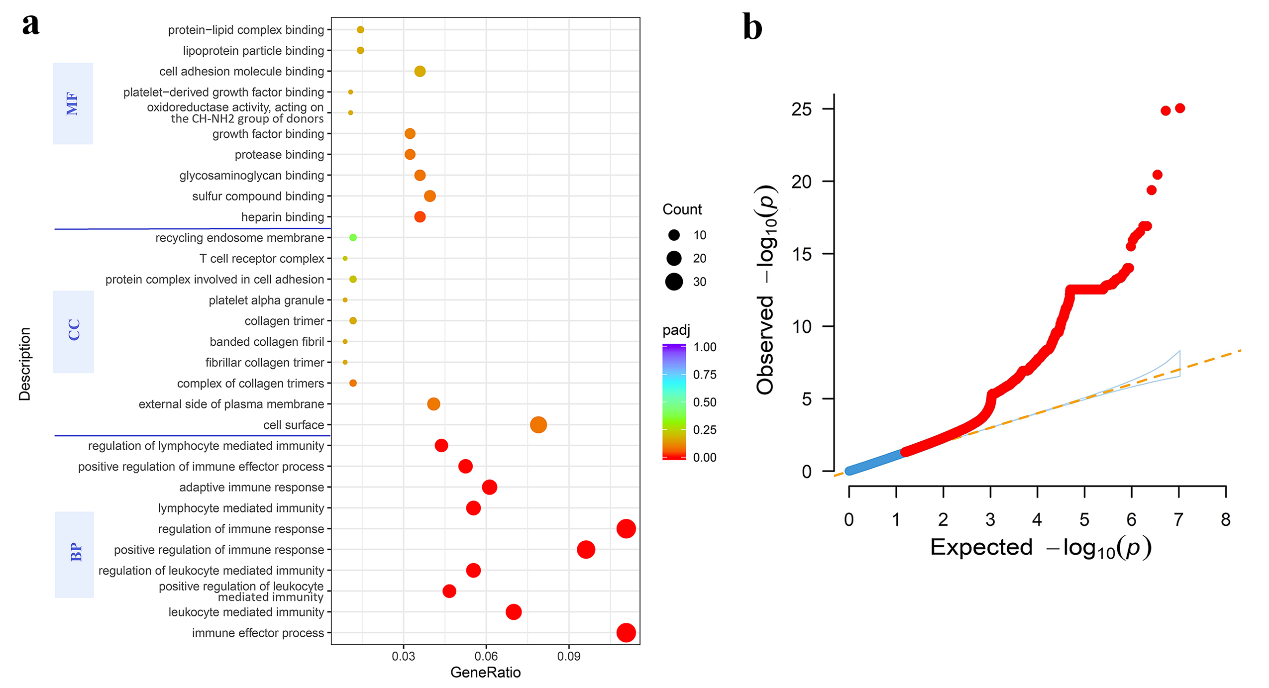


Figure S2 Critical genes in the intramuscular fat (IMF) content-related midnightblue and darkgreen modules.

(a) Identification of critical genes in midnightblue module based on *p*-values of gene significance (GS) and module membership (MM). (b) Protein-protein interaction (PPI) network for the critical genes in midnightblue module. (c) Identification of critical genes in darkgreen module based on p-values of GS and MM. (d) PPI network for the critical genes in darkgreen module.


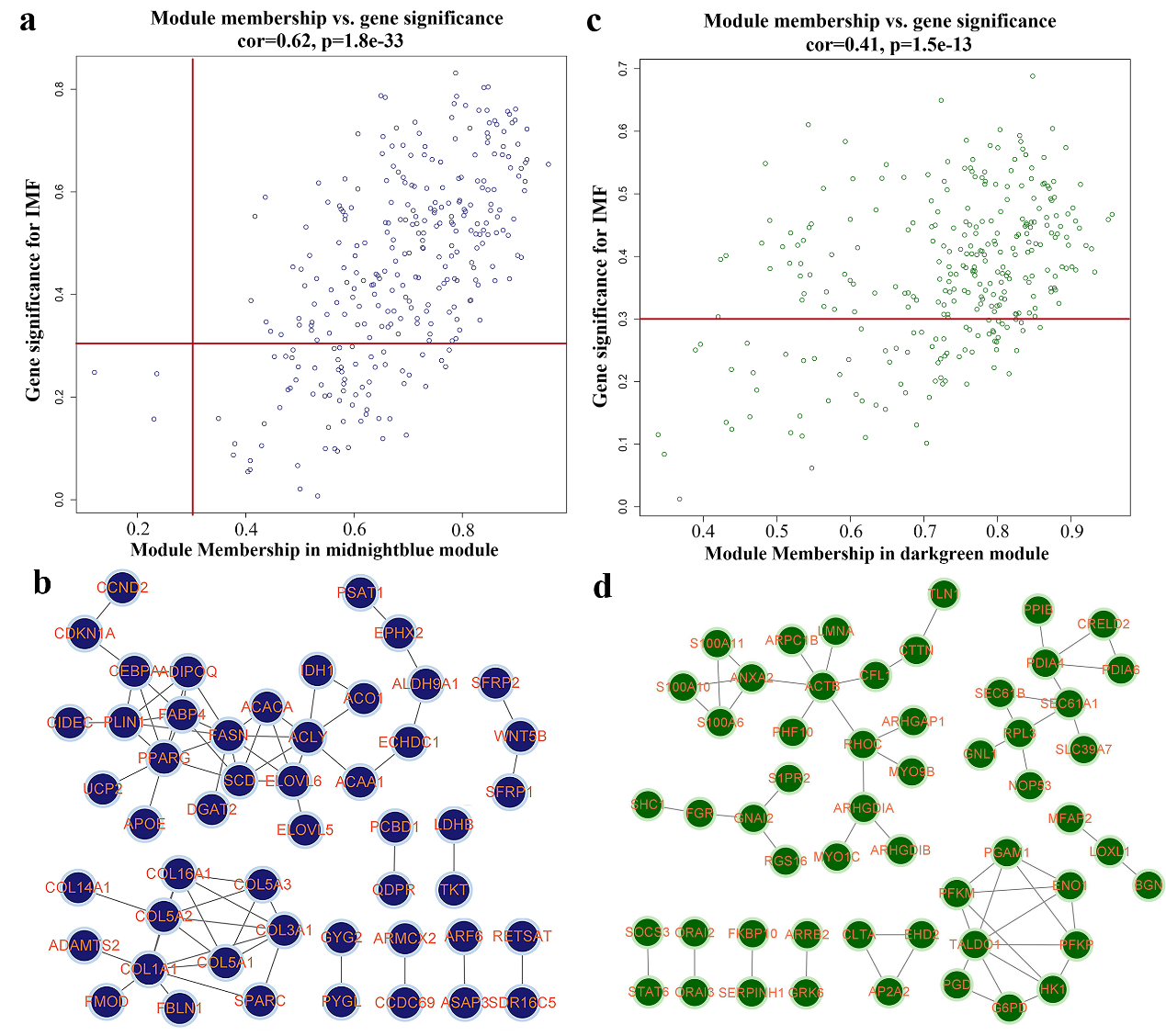

Supplement: Supplementary file 3 — Supplementary Material 3. [file 12711_2025_1014_MOESM3_ESM.docx]
